# Self-reported lactose intolerance is inversely associated with calcium intake and bone mineral density: a cross-sectional data analysis from the Iwaki Health Promotion Project

**DOI:** 10.1007/s00394-025-03856-x

**Published:** 2025-12-06

**Authors:** Daisuke Kawata, Ayatake Nakano, Hiroshi M. Ueno, Yota Tatara, Eiji Sasaki, Yasuyuki Ishibashi, Yoshinori Tamada, Tatsuya Mikami, Koichi Murashita, Shigeyuki Nakaji, Ken Itoh

**Affiliations:** 1 Department of Precision Nutrition for Dairy Foods, Hirosaki University Graduate School of Medicine, 5 Zaifu-Cho, Hirosaki, Aomori, 036-8562 Japan; 2 Milk Science Research Institute, Megmilk Snow Brand Co., Ltd., Kawagoe, Japan; 3 Biomedical Research Center, Hirosaki University Graduate School of Medicine, Hirosaki, Japan; 4 Department of Orthopaedic Surgery, Hirosaki University Graduate School of Medicine, Hirosaki, Japan; 5 Department of Medical Data Intelligence, Research Center for Health-Medical Data Science, Hirosaki University Graduate School of Medicine, Hirosaki, Japan; 6 Department of Preemptive Medicine, Innovation Center for Health Promotion, Hirosaki University Graduate School of Medicine, Hirosaki, Japan; 7 Research Institute of Health Innovation, Hirosaki University, Hirosaki, Japan; 8 Department of Stress Response Science, Biomedical Research Center, Hirosaki University Graduate School of Medicine, Hirosaki, Japan

**Keywords:** Lactose intolerance, Dairy foods, Calcium, Bone mineral density, Iwaki Health Promotion Project

## Abstract

**Purpose:**

Self-reported lactose intolerance (LI) is associated with gastrointestinal symptoms after consuming lactose-containing foods and avoiding dairy products; however, few studies have investigated its prevalence and nutritional significance in Asian populations. Consequently, this study aimed to determine the relationship between self-reported LI and bone health in Japanese adults.

**Methods:**

A community-based nutritional survey, including self-reported LI and forearm bone mineral density (BMD) measurements, was conducted. In 843 healthy Japanese adults, as part of an annual medical checkup at the Iwaki Health Promotion Project 2023, self-reported LI prevalence was characterized based on the presence of subjective abdominal symptoms after consuming lactose-containing foods. Relationships among self-reported LI, dairy consumption, calcium intake, and BMD were analyzed.

**Results:**

Participants with the self-reported LI (n = 191, 22.7% of the participants) had lower intakes of milk and calcium than those without self-reported LI (n = 652; 32.9 ± 83.8 vs. 63.4 ± 111.9 g/1000 kcal/d for milk and 243.7 ± 140.4 vs. 285.5 ± 162.3 mg/1000 kcal/d for calcium, respectively). The presence of self-reported LI was associated with the decreased BMD Z-score after adjustments with age, sex, body mass index, smoking, alcohol, vitamin D, and walking speed (β = –0.1615, *P* = 0.042), and comparison using propensity score matching revealed that self-reported LI was associated with a significant decrease in BMD parameters after the adjustments (β =  −  0.2543, − 0.3903, and − 0.0148; *P* = 0.007, 0.010, and 0.023 for Z-score, T-score, and BMD itself, respectively).

**Conclusion:**

Self-reported LI is negatively associated with calcium intake and bone health in healthy Japanese adults.

**Clinical trial registration number:**

UMIN000040459 (https://center6.umin.ac.jp/cgi-open-bin/ctr/ctr_view.cgi?recptno=R000046175). Registration date: May 20, 2020.

**Supplementary Information:**

The online version contains supplementary material available at 10.1007/s00394-025-03856-x.

## Introduction

Lactose intolerance (LI) is characterized by an inability to completely digest lactose, primarily owing to lactase deficiency, whereas lactose malabsorption occurs when lactose is not easily digested due to low lactase levels. Lactase non-persistence reduces the production of lactase after infancy and involves varying degrees of LI due to lactose malabsorption when lactose-containing dairy foods are consumed [1, 2]. These concepts are interrelated and complex in terms of understanding biological conditions, clinical relevance, and personal experiences.

Self-reported LI presents a slightly different concept in LI-related conditions that aligns with personal experiences and subjective perceptions of lactose digestion ability. Several individuals may report symptoms of LI after consuming dairy products, even if they are not clinically diagnosed with LI, and may tolerate small amounts of lactose with no physiological issues [3, 4]. Self-reported LI results in lower calcium intake and correlated with hypertension and diabetes in American adults [3], lower bone mineral density (BMD) in Slovakian young adults [5], and quality of life and food choices not limited to dairy products in Chinese adults [6].

Calcium is an essential nutrient for bone health, and its insufficiency decreases BMD, leading to osteoporosis [7–9]. Dairy products, including milk, yogurt, and cheese, are abundant in calcium and are major sources of calcium for humans [10]. Adequate intake of dairy products is recommended to maintain bone health and prevent osteoporosis [11, 12]. Dairy calcium levels are influenced by the dietary patterns across different regions. Therefore, the effect of LI on calcium intake and BMD should be examined in each specific region. For example, the mean intake of dairy products by Japanese adults is approximately 110 g/day, relatively lower than that in Western countries [13–16]. Behavioral avoidance of dairy products is likely to be more affected by subjective symptoms than by a definitive diagnosis; hence, it is important to investigate the relationship between self-reported LI and nutritional intake or bone health. The main causes of symptoms of LI are lactose in milk. Therefore, low-lactose dairy products may serve as an adjunctive intervention alongside non-dairy calcium intake [17]. Low-lactose milk can be produced through lactose hydrolysis or membrane filtration, providing a straightforward approach to supply dairy calcium for individuals with self-reported LI [18].

However, to the best of our knowledge, there have been no studies on the relationship among self-reported LI, calcium intake, and bone health in a large community-based setting, and it is significant to investigate this in Asian populations, which have a high prevalence of LI [19]. Consequently, a cross-sectional study was conducted on the relationship between self-reported LI and bone health, including calcium intake and BMD, in Japanese adults. A dietary survey was conducted, and BMD was measured as part of the Iwaki Health Promotion Project (IHPP) 2023, a large-scale annual medical checkup program [20]. Furthermore, a nutritional survey on the dietary habits of low-lactose milk was conducted. Individuals with LI should be encouraged to limit, rather than eliminate, lactose consumption to optimize the nutritional benefits of dairy products [21]. Hence, the awareness and intake frequency of low-lactose milk were also investigated.

## Materials and methods

### Study design

Data from the IHPP 2023 were analyzed in this cross-sectional study. The IHPP is a community-based annual health checkup program conducted on approximately 1,000 residents of Hirosaki City in Japan. The IHPP began in 2005 and now encompasses approximately 3000 items related to body health. In this study, basic demographics, such as age, body weight, and height, physical checkup data, and lifestyle surveys, including smoking status, BMD, and dietary survey results, were analyzed cross-sectionally [20].

### Ethics approval

This study was conducted in accordance with the 1964 Declaration of Helsinki and its later amendments and was approved by the Internal Review Board of Hirosaki University (ref.: 2023-007-1 and HCN2024-1-0408, approved on May 2, 2023, and April 8, 2024, respectively). The study was registered in the Japanese Clinical Trials Registry (UMIN000040459, https://center6.umin.ac.jp/cgi-open-bin/ctr/ctr_view.cgi?recptno=R000046175). All included participants provided written informed consent at the time of enrolment in the IHPP 2023 in June 2023, and the opt-out procedure for this study was completed in April 2024.

### Study participants

**Fig. 1:**
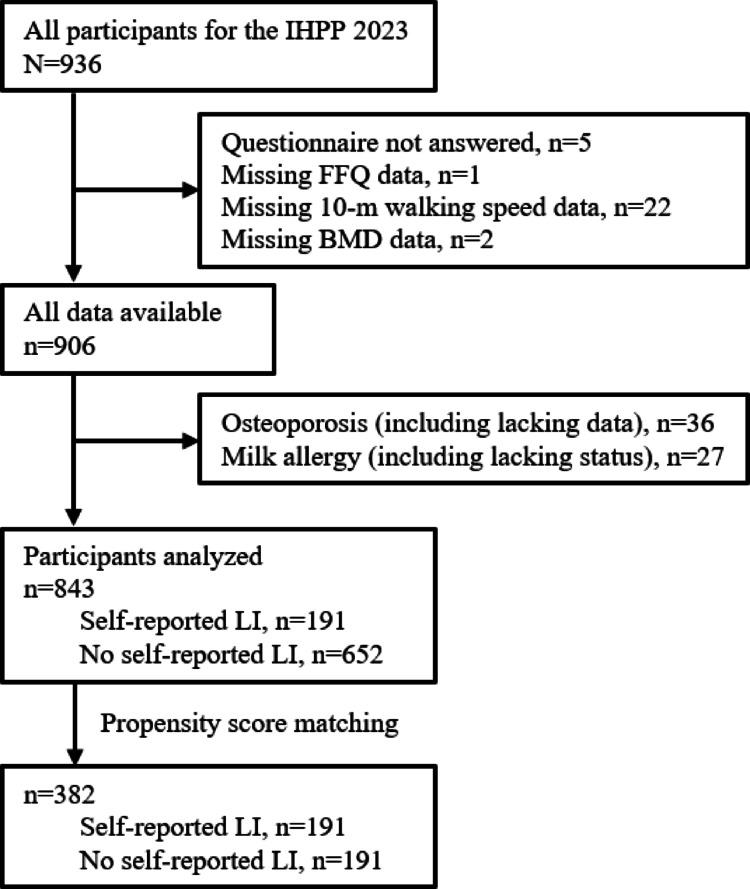
Flowchart of participant recruitment and grouping. IHPP 2023 recruited 936 participants, and 843 participants were analyzed. After propensity score matching, 382 participants were analyzed

In total, 936 adult residents from the Iwaki region (Hirosaki City, Aomori, Japan) were recruited for the IHPP 2023. Among them, five participants with missing data on the LI survey, one with missing data on the food frequency questionnaire (FFQ), 22 with missing data on walking speed, and two with outliers or missing BMD data were excluded from this study. Of these, 36 patients with a documented history of diagnosed osteoporosis or with insufficient medical history due to incomplete or invalid responses in the questionnaire, along with 27 individuals with a milk allergy or with ambiguous milk allergy status due to incomplete or invalid questionnaire responses, were excluded. Finally, 843 participants were included in this study (Fig. 1).

#### Sociodemographic and lifestyle questionnaires

Participants for the IHPP 2023 were required to provide responses to the questionnaires in advance of the IHPP 2023 visit, including sociodemographic, lifestyle, FFQ, medical history, and other interview items. The participants were interviewed face-to-face about unclear responses and unanswered questions on the day of the medical checkup.

#### Questionnaire for self-reported lactose intolerance

The participants were instructed to respond to the question, “After drinking milk, do you experience uncomfortable symptoms, such as a rumbling stomach, a distension stomach, feeling sick, or diarrhea?” from five options: “always,” “often,” “sometimes,” “almost never,” or “not at all.” The participants who answered “always,” “often,” or “sometimes” were classified as having self-reported LI. The participants who answered “almost never” or “not at all” were classified as having no self-reported LI. The participants grouped according to self-reported LI were also asked about the nature of their symptoms after consuming milk.

#### Questionnaire for attitudes toward calcium intake and calcium sufficiency

The participants were instructed to respond to the question, “Do you try to take calcium in your daily life and diet?” from five options: “always,” “often,” “sometimes,” “not much,” or “No.” and the question, “Do you think you are getting enough calcium in your daily life?” from five option: “sufficient,” “almost,” “some extent,” “not much,” and “No.”

#### Questionnaire for the awareness and consumption of low-lactose milk

The participants were instructed to respond to the question “Do you know low-lactose milk (milk that does not cause stomach rumbling)?” from three options: “Yes,” “I’ve heard of it, but don’t know much about it,” and “No.” The participants who answered “Yes” were further questioned regarding their consumption frequency of low-lactose milk from the following 7 options: “Every day,” “5–6 days per week,” “3–4 days per week,” “1–2 days per week,” “1–3 days per month,” “Less than 1 day per month,” and “Never had a drink.”

#### Food frequency questionnaire

The FFQ (long version) provided by the Education Software (Tokyo, Japan) was employed to measure dietary and nutritional intake. The FFQ was designed based on the Japan Public Health Center-based Prospective Study for the Next Generation, which is a cohort study on the relationship among Japanese lifestyle, environment, and disease [22]. The FFQ consists of 185 questions and can be used to estimate the daily dietary and nutritional intake status using a single survey. The FFQ was validated using a 12-day weighed food record. For milk and calcium intake, Spearman’s correlation coefficients with 12-day weighed food records were 0.42–058 [22].

#### Anthropometrics and 10-m walking speed

Anthropometrics, such as height and weight, were measured during the medical checkup, and BMI was calculated from these data. A 10-m walking speed test was performed using a previously described procedure [23]. Briefly, the participants were instructed to walk at their normal speed for 14 m, with the time recorded for the central 10 m. The 10-m walking speed was calculated in m/s.

#### Bone mineral density

The BMD of the radius was measured utilizing dual-energy X-ray absorptiometry using DCS-600EXV (Hitachi Aloka Medical, Tokyo, Japan) following a previously reported method [24, 25]. The BMD at one-third of the distal radius on the nondominant side was measured unless there was a history of fracture, in which case the dominant side was measured. BMD, Z-, and T-scores were used for further analysis. The Z-score is the value of the deviation obtained when compared with the mean value of age and sex-matched standards. The T-score is the value obtained when compared with the mean value of a younger reference group.

### Statistical analyses

Group comparisons of self-reported and non-self-reported LI participants were performed using the chi-square test for categorical variables and the Mann–Whitney *U* test for continuous variables.

An adjusted multivariate linear regression analysis was performed to identify the impact of self-reported LI on BMD. For all participants, the three models were analyzed using the forced entry method. Bone health, or BMD, is affected by age, sex, BMI, and other lifestyle factors, such as smoking habits, alcohol intake, and vitamin D status [26–34]. Moreover, walking speed, which represents exercise capacity, is also associated with bone density [35, 36]. In Model 1, simple adjustments for age, sex, and BMI were performed. As smoking and alcohol consumption are well-established risk factors for fractures [26–29] and may reduce BMD [30, 31], smoking status and alcohol intake were included in Model 2. Walking speed as motor capacity is related to BMD in older women [35, 36], and vitamin D is critical for bone health [32, 33]; thus, these factors were included in Model 3.

The associations with BMD indices were then examined after propensity score matching to minimize the influence of confounding factors, including age, sex, and BMI. Thus, age, sex, and BMI were set as cofactors, and the propensity score was calculated. Each participant with self-reported LI was matched to non-self-reported LI counterparts. The difference in the propensity scores allowed for matching was set to 20% of the standard deviation of the entire set of propensity scores. After matching, an adjusted multivariate linear regression analysis was performed to identify the impact of self-reported LI on BMD using the adjustment factors of Model 3. As BMD itself and BMD T-scores decline rapidly from approximately 50 years, age^2^ was used as an adjustment factor instead of age for BMD and BMD T-scores.

Analyses were performed using Python 3.11.4. Statistical significance was set at *P* < 0.05 and adjusted using the Bonferroni correction for multiple comparisons.

## Results

**Fig. 2:**
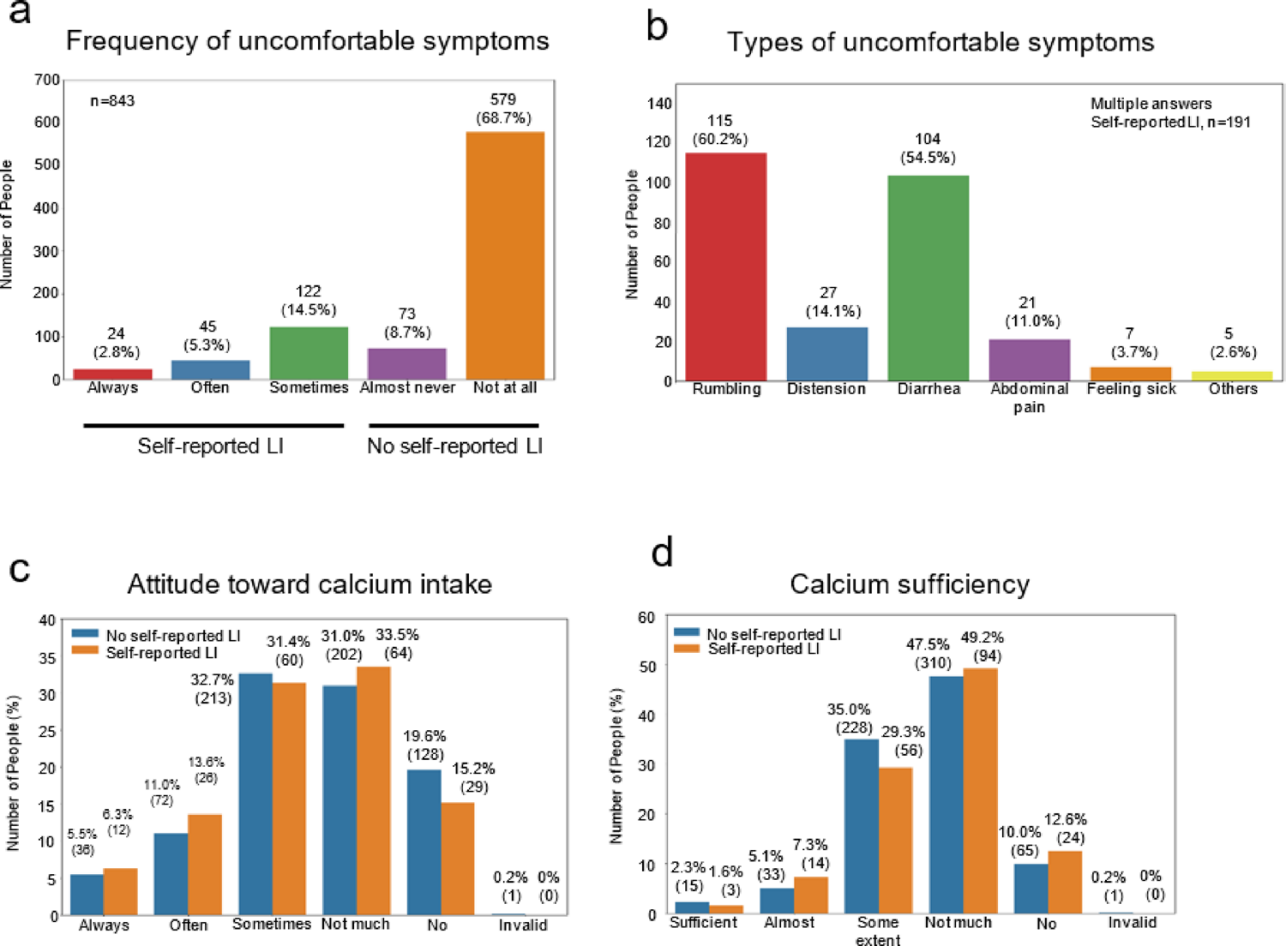
The participants were asked, “After drinking milk, do you experience uncomfortable symptoms, such as a rumbling stomach, a stomach distension, feeling sick, or diarrhea?” (**a**). The participants grouped according to self-reported LI were also asked about the nature of their symptoms after consuming milk (**b**). n (%). The participants were also asked, “Do you try to take calcium in your daily life and diet?” (**c**) and “Do you think you are getting enough calcium in your daily life?” (**d**). % (n)

**Fig. 3:**
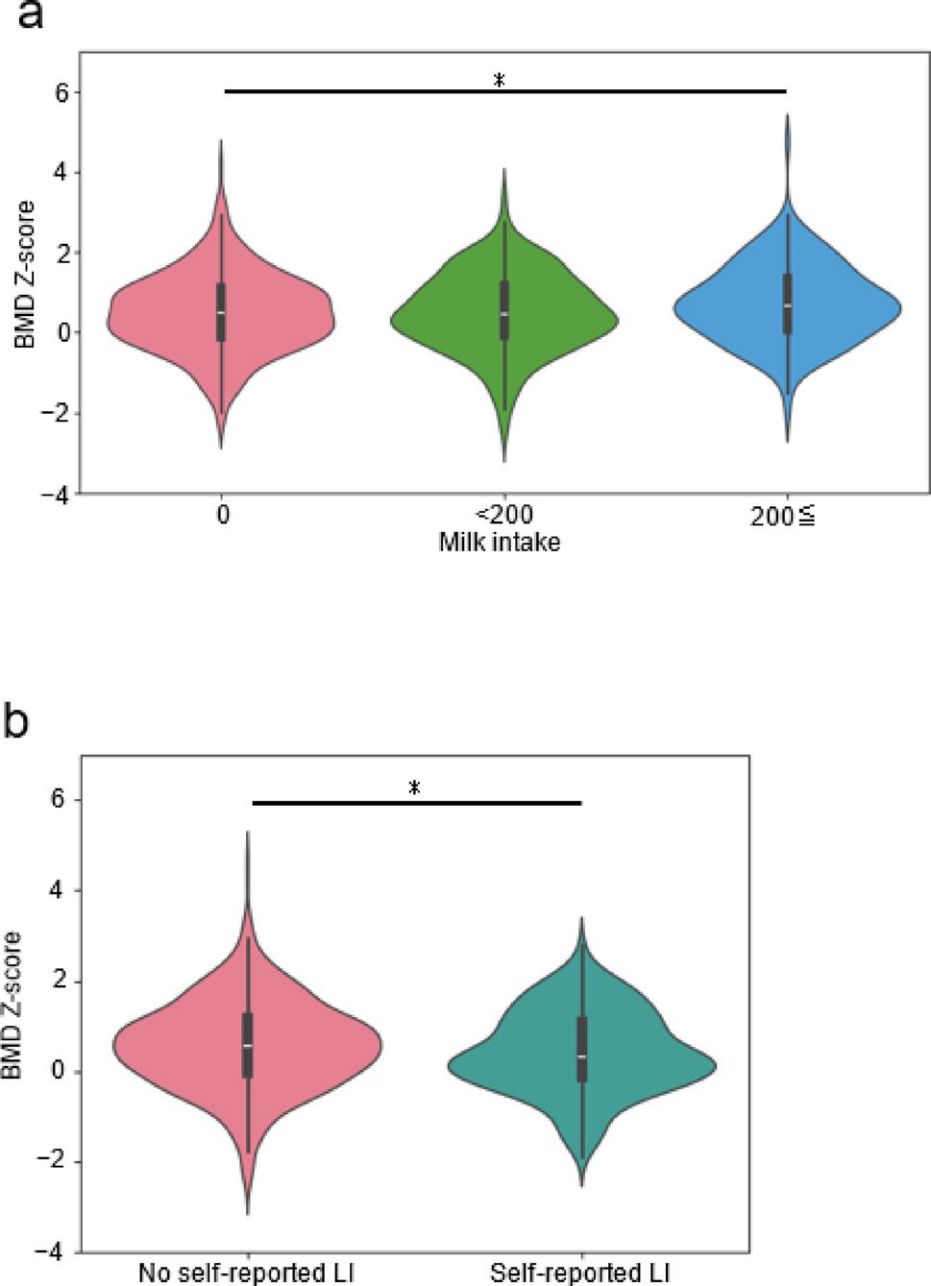
The participants in the Iwaki Health Promotion Project 2023 were grouped by calcium intake (**a**) or presence of self-reported lactose intolerance (**b**). The Mann–Whitney *U* test and the Bonferroni correction were performed. *P < 0.05. BMD, bone mineral density; LI, lactose intolerance

Among the 843 participants, 191 had self-reported LI, and 652 had no self-reported LI (Fig. 2a, Table 1). Regarding the type of abdominal symptoms, > 50% of the participants with self-reported LI reported stomach rumbling or diarrhea (Fig. 2b). No significant differences in sex or age were observed between the two groups. The ages of participants with self-reported LI ranged from 21 to 84 years, whereas those without self-reported LI ranged from 20 to 87 years. Milk consumption of the self-reported LI group was significantly lower than that of the non-self-reported LI group, whereas yogurt consumption was of borderline significance, and cheese consumption was comparable between the two groups. No differences were found in the intake of other main calcium sources in the Japanese diet, such as fish, legumes, or vegetables [37]. Although no significant difference was found between the two groups in their attitudes toward calcium intake and sense of calcium sufficiency (the chi-square test; *P* = 0.570 and *P* = 0.385, respectively) (Fig. 2c, 2d), the calcium intake in the self-reported LI group was significantly lower than that in the non-self-reported LI group (Table 1). The BMD Z-score was significantly higher in participants consuming milk > 200 g/day than in participants not consuming milk daily; moreover, it was significantly lower in individuals with self-reported LI than in non-self-reported LI (Fig. 3a, 3b).

**Table 1:** Baseline and demographic characteristics of the participants with and without self-reported lactose intolerance in the Iwaki Health Promotion Project 2023

	Total	Self-reported LI	No self-reported LI	*P*-value
Variables	(N = 843)	(n = 191)	(n = 652)	
*Demographic characteristics*				
Age (year)	51.5 ± 14.1	49.9 ± 13.4	51.9 ± 14.2	0.07
Sex (male, n [%])	373 (44.2%)	108 (43.5%)	290 (44.5%)	0.87
BMI (kg/m^2^)	23.1 ± 3.6	22.9 ± 3.3	23.1 ± 3.6	0.63
Smoking status (n [%])				< 0.05
Current	155 (18.4%)	30 (15.7%)	125 (19.2%)	
Former	280 (33.2%)	77 (40.3%)	203 (31.1%)	
Never	408 (48.4%)	84 (44.0%)	324 (49.7%)	
Walking speed [m/sec]	1.4 ± 0.2	1.4 ± 0.2	1.4 ± 0.2	0.52
*Foods and nutrients*				
Energy [kcal/day]	2199.4 ± 1046.1	2114.2 ± 1233.9	2224.3 ± 984.0	< 0.01
Protein [g/day]	74.6 ± 42.5	71.6 ± 50.4	75.5 ± 39.9	< 0.05
[g/1000 kcal/day]	33.5 ± 6.8	33.3 ± 6.7	33.5 ± 6.8	0.60
Fat [g/day]	69.5 ± 45.4	68.7 ± 61.4	69.7 ± 39.6	< 0.05
[g/1000 kcal/day]	30.9 ± 8.6	31.1 ± 8.7	30.8 ± 8.5	0.56
Carbohydrate [g/day]	279.0 ± 139.0	264.0 ± 129.9	283.4 ± 141.3	< 0.001
[g/1000 kcal/day]	129.4 ± 23.5	128.9 ± 22.5	129.5 ± 23.8	0.54
Milk [g/day]	139.0 ± 326.9	84.1 ± 276.0	155.0 ± 338.9	< 0.001
[g/1000 kcal/day]	56.5 ± 106.9	32.9 ± 83.8	63.4 ± 111.9	< 0.001
Yogurt [g/day]	76.9 ± 197.5	61.9 ± 179.5	81.2 ± 202.4	< 0.05
[g/1000 kcal/day]	30.9 ± 64.3	25.4 ± 59.8	32.6 ± 65.5	0.06
Cheese [g/day]	4.4 ± 10.6	4.7 ± 12.6	4.3 ± 9.9	0.65
[g/1000 kcal/day]	2.1 ± 4.3	2.2 ± 4.3	2.0 ± 4.3	0.98
Peas (including soybeans) [g/day]	77.0 ± 99.4	84.9 ± 113.1	74.7 ± 95.0	0.34
[g/1000 kcal/day]	35.3 ± 42.5	42.1 ± 58.7	33.3 ± 36.2	0.07
Green and yellow vegetables [g/day]	94.1 ± 99.5	87.3 ± 91.7	96.1 ± 101.6	0.42
[g/1000 kcal/day]	44.2 ± 46.4	41.5 ± 37.2	45.0 ± 48.7	0.85
Fish [g/day]	48.1 ± 47.0	46.1 ± 48.8	48.7 ± 46.5	0.60
[g/1000 kcal/day]	21.2 ± 16.0	21.3 ± 15.1	21.2 ± 16.3	0.74
Alcohol [g/day]	18.7 ± 32.3	17.5 ± 29.2	19.0 ± 33.1	0.91
[g/1000 kcal/day]	8.2 ± 13.3	8.1 ± 13.1	8.2 ± 13.4	0.83
Calcium [mg/day]	632.0 ± 587.6	538.7 ± 590.5	659.3 ± 584.4	< 0.001
Calcium [mg/1000 kcal/day]	276.0 ± 158.5	243.7 ± 140.4	285.5 ± 162.3	< 0.001
Vitamin D [μg/day]	6.8 ± 6.3	6.5 ± 6.3	6.9 ± 6.4	0.19
[μg/1000 kcal/day]	3.0 ± 2.1	3.0 ± 2.1	3.0 ± 2.0	0.88

Self-reported LI, self-reported lactose intolerance

Data are presented as mean ± standard deviation or n [%]

Bone health, or BMD, is affected by age, sex, BMI, and other lifestyle factors, including smoking habits, alcohol intake, and vitamin D intake [26–34]. Moreover, walking speed, which represents an individual’s exercise capacity, is associated with bone density [35, 36]. Therefore, multiple regression models were developed with further adjustments. In all the models, self-reported LI was significantly associated with lower BMD Z-scores after adjusting for these factors (Table 2). Furthermore, multiple regression analysis after propensity score matching revealed that self-reported LI was associated with lower BMD Z-scores and lower BMD and BMD T-scores (Table 3). All standardized mean differences of the matched cofactors (age, sex, BMI) were < 0.1 (Supplementary Fig. 1).

**Table 2:** Association of self-reported lactose intolerance with bone mineral density Z-score among participants of the Iwaki Health Promotion Project 2023

	Model 1				Model 2				Model 3			
Variables	β	95% CI	*P*		β	95% CI	*P*		β	95% CI	*P*	
Self-reported LI (“Yes” = 1, “No” = 0)	− 0.1576	(− 0.312, − 0.003)	0.046	*	− 0.1642	(− 0.320, − 0.009)	0.038	*	− 0.1615	(− 0.317, − 0.006)	0.042	*
Age (year)	− 0.0028	(− 0.007, 0.002)	0.233		− 0.0031	(− 0.008, 0.002)	0.197		− 0.0027	(− 0.007, 0.002)	0.271	
Sex (“Male” = 0, “Female” = 1)	0.4196	(0.285, 0.555)	< 0.001	***	0.4553	(0.307, 0.603)	< 0.001	***	0.4588	(0.311, 0.607)	< 0.001	***
BMI (kg/m^2^)	0.0270	(0.008, 0.046)	0.005	**	0.0271	(0.008, 0.046)	0.005	**	0.0286	(0.010, 0.048)	0.003	**
Smoking status (current) (“Yes” = 1, “No” = 0)					0.0208	(− 0.170, 0.211)	0.831		0.0237	(− 0.167, 0.214)	0.808	
Smoking status (former) (“Yes” = 1, “No” = 0)					0.0721	(− 0.085, 0.229)	0.369		0.0749	(− 0.082, 0.232)	0.350	
Alcohol consumption (g/1000 kcal/day)					0.0021	(− 0.003, 0.007)	0.424		0.0021	(− 0.003, 0.007)	0.439	
Vitamin D (μg/1000 kcal/day)									− 0.0111	(− 0.043, 0.021)	0.501	
Walking speed (m/sec)									0.3470	(− 0.006, 0.701)	0.054	
Const	− 0.1283	(− 0.643, 0.386)	0.624		− 0.1787	(− 0.704, 0.347)	0.505		− 0.7049	(− 1.472, 0.062)	0.072	

Self-reported LI, self-reported lactose intolerance; CI, confidence interval; β, partial regression coefficient

*P < 0.05, ** P < 0.01, *** *P* < 0.001

**Table 3:** Association of self-reported lactose intolerance with bone mineral density after propensity score matching among participants of the Iwaki Health Promotion Project 2023

Objective variable	Z-score				T-score				Bone mineral density (g/cm^2^)			
Variables	β	95% CI	*P*		β	95% CI	*P*		β	95% CI	*P*	
Self-reported LI (“Yes” = 1, “No” = 0)	− 0.2543	(− 0.440, − 0.069)	0.007	**	− 0.3093	(− 0.545, − 0.073)	0.010	*	− 0.0148	(− 0.028, − 0.002)	0.023	*
Age (year)	− 0.0030	(− 0.010, 0.004)	0.406									
Age^2^ /100 (year^2^/100)					− 0.0638	(− 0.073, − 0.055)	< 0.001	***	− 0.0035	(− 0.004, − 0.003)	< 0.001	***
Sex (“Male” = 0, “Female” = 1)	0.5083	(0.292, 0.725)	< 0.001	***	− 0.1574	(− 0.433, 0.118)	0.262		− 0.1285	(− 0.143, − 0.114)	< 0.001	***
BMI (kg/m^2^)	0.0387	(0.011, 0.067)	0.007	**	0.0264	(− 0.009, 0.062)	0.144		0.0018	(0.000, 0.004)	0.059	
Smoking status (current) (“Yes” = 1, “No” = 0)	− 0.0569	(− 0.328, 0.214)	0.680		0.0515	(− 0.294, 0.396)	0.769		0.0009	(− 0.018, 0.020)	0.926	
Smoking status (former) (“Yes” = 1, “No” = 0)	0.0427	(− 0.183, 0.269)	0.710		0.1965	(− 0.090, 0.483)	0.178		0.0107	(− 0.005, 0.026)	0.176	
Alcohol consumption (g/1000 kcal/day)	0.0113	(0.003, 0.020)	0.011	*	0.0138	(0.003, 0.025)	0.015	*	0.0021	(0.000, 0.001)	0.021	*
Vitamin D (μg/1000 kcal/day)	− 0.0135	(− 0.062, 0.035)	0.586		− 0.0259	(− 0.088, 0.036)	0.414		− 0.0012	(− 0.005, 0.002)	0.501	
Walking speed (m/sec)	0.3798	(− 0.116, 0.876)	0.133		0.1480	(− 0.483, 0.779)	0.645		0.0164	(− 0.018, 0.051)	0.344	
Const	− 0.9413	(− 2.041, 0.158)	0.093		0.9053	(− 0.455, 2.265)	0.191		0.7941	(0.721, 0.868)	< 0.001	***

Self-reported LI, self-reported lactose intolerance; CI, confidence interval; β, partial regression coefficient

*P < 0.05, ** P < 0.01, *** *P* < 0.001

A subgroup analysis was conducted. Participants were categorized into three age groups: under 45 years, 45–60 years, and over 60 years, and analyzed by gender. Particularly in women, hormonal balance changes during the menopause period, typically around the age of 50, leading to a decrease in BMD. As a result, self-reported LI demonstrated a significant inverse association with BMD Z-score, among females under 45, individuals aged 45–60 years, and males aged 45–60 years (Supplementary Table 1).

**Fig. 4:**
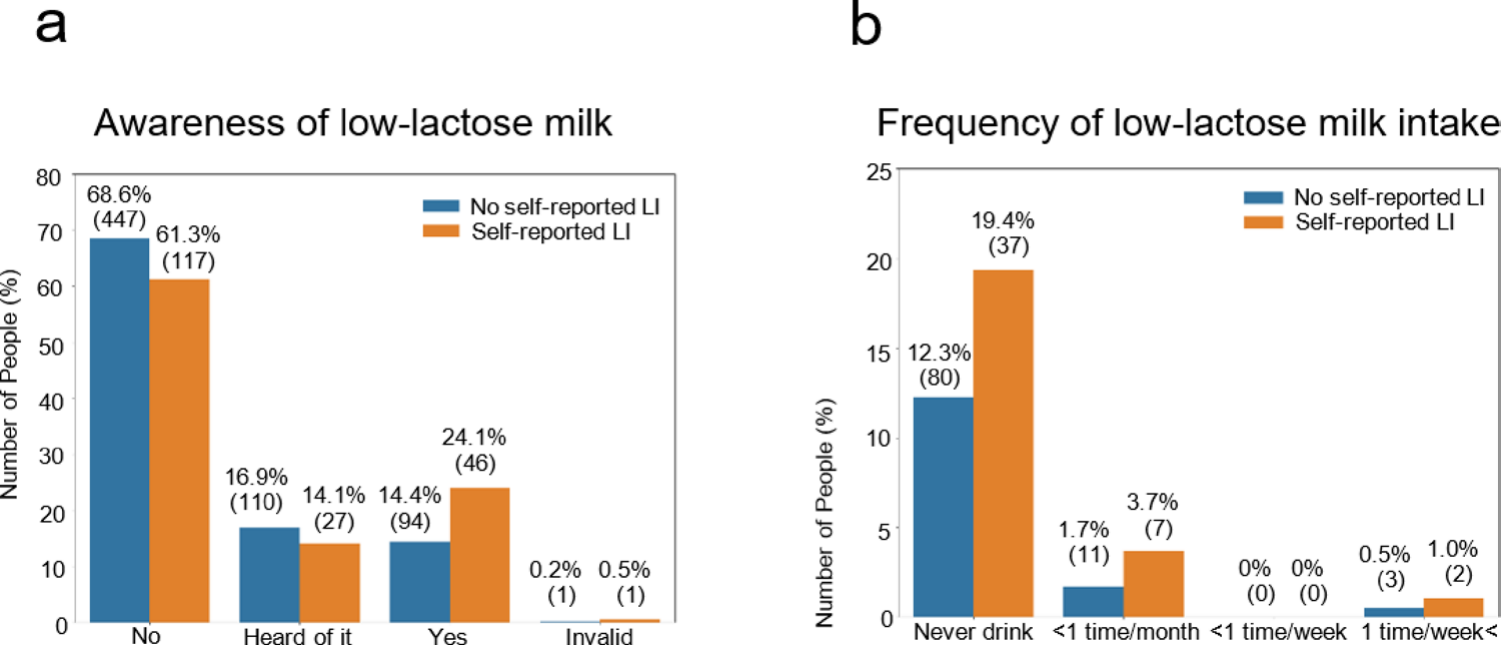
The participants were asked, “Do you know low-lactose milk (milk that does not cause stomach rumbling)?” (**a**). The participants who answered “Yes” were further questioned regarding their consumption frequency of low-lactose milk (**b**). % (n)

Low-lactose milk is well tolerated by lactose-intolerant populations [38]; thus, low-lactose milk is a promising option for calcium supplementation and maintenance of BMD in self-reported LI populations. Given that lactose-hydrolyzed milk has been available in the Japanese market for approximately 50 years, participants were questioned about their awareness of low-lactose milk and the frequency of its consumption. The results revealed that 30–40% of the participants were aware of low-lactose milk, and < 5% consumed this milk more than once a month, regardless of self-reported LI status (Fig. 4).

## Discussion

In this study, Japanese adults with self-reported LI revealed lower milk consumption and calcium intake than those with non-self-reported LI. A significant inverse association between self-reported LI and BMD Z-scores was observed in some groups during the subgroup analysis. In females, a significant relationship was observed in the younger cohort. This is probably due to this group being less influenced by hormonal changes, which exhibit significant individual differences. Furthermore, the exclusion of individuals diagnosed with osteoporosis from the analysis may have introduced bias, particularly impacting the older age demographic. On the other hand, the analysis that included all participants with self-reported LI had a lower BMD Z-score, indicating that the BMD of individuals with self-reported LI was significantly lower than that of their age-matched counterparts without self-reported LI. Propensity score matching further revealed that, besides the BMD Z-score, self-reported LI was associated with BMD and a lower BMD T-score (in comparison with the young group), suggesting that self-reported LI is associated with milk avoidance, reduced calcium intake, and a decline in BMD.

The intake of calcium and vitamin D supplements could have potentially affected the outcome of the results obtained. Nevertheless, this study did not account for supplement usage because the total number of regular calcium supplement users was only 17 (2.0%), which included three individuals from the self-reported LI group (1.6%). Additionally, there were 40 users (4.7%) of supplements containing vitamin D, with seven individuals from the self-reported LI group (3.7%). Furthermore, Fisher’s exact test revealed no significant differences between groups in the number of users for each supplement. In fact, incorporating supplement usage as an adjustment factor had little effect on the results (Supplementary Table 2).

The prevalence of individuals with self-reported LI in the study population was 22.7%, which was 3.2% higher than that of African Americans in a multiethnic study [39]. Previous studies have reported inconsistent results regarding the association among LI, calcium intake, and BMD. For example, studies in Turkey and Thailand have reported that LI or LI symptoms cause a significant decrease in calcium intake and BMD [40, 41]. However, other studies on German–Turkish immigrants and the United States have not found a significant relationship between LI and BMD [42, 43]. One possible explanation is the diverse dietary patterns of various geographic and ethnic regions. The amount and type of dairy food consumed vary, and the source of nondairy calcium differs according to ethnic traits [44]. Calcium from natural and tap water accounts for the dietary calcium; however, the aqueous calcium content varies geographically [45, 46]. This study found no difference in the intake of legumes, vegetables, and fish as nondairy dietary calcium sources between the participants with or without self-reported LI in the Japanese population [37, 47]. The difference in mean calcium intake between the self-reported LI group and the non-self-reported LI was approximately 42 mg/1000 kcal/day. The differences in mean milk and yogurt intake were 30 and 7 g/1000 kcal/day, and their total was equivalent to 41 mg/1000 kcal/day of calcium [48]. Accordingly, dietary avoidance of milk owing to abdominal symptoms in self-reported LI appears to be associated with decreased calcium intake and BMD in the Japanese population. Furthermore, the fact that the trends in attitude toward calcium intake and sense of calcium sufficiency did not differ suggests that the decline in calcium intake occurs unconsciously.

Fractures are associated with short life expectancy and poor quality of life in older adults [49, 50], and bone health is important for a healthy life expectancy. In particular, Japan has one of the longest life expectancies worldwide [51], and maintaining quality of life throughout life is a major challenge. Our data suggest that calcium supplementation in individuals with self-reported LI is important for preventing BMD decline and osteoporosis. Ensuring adequate calcium levels from various sources can help maintain bone health [17].

In a typical Japanese diet, soy products, fish, and green and yellow vegetables are rich in calcium [37]. Encouraging individuals with self-reported LI to consume such foods or promoting the intake of calcium supplements is advisable. Aged cheese and cultured yogurt, although derived from milk, contain reduced lactose levels and are therefore recommended [17]. In addition, low-lactose milk contains an equivalent calcium content to that of typical Japanese soy product, Tofu, and has similar nutritional benefits to regular milk [52]. Low-lactose milk can be conveniently consumed; hence, in addition to promoting the intake of nondairy calcium, drinking low-lactose milk is a promising option to increase dairy calcium intake in self-reported LI. The availability of low-lactose dairy products (including lactose-free dairy products) has increased in the last decade because of the growing demand for these products among the lactose-intolerant populations [53], and the consumption of low-lactose milk is recommended for the prevention of osteoporosis in LI populations [54, 55]. Although lactose-hydrolyzed milk are widely available in Japan, awareness and consumption of low-lactose milk remain low. This can be attributed to the insufficient awareness regarding the differences between regular milk and low-lactose milk, as well as the recent increase in plant-based milk, including soybean milk, pea milk, oat milk, and almond milk, as dairy alternatives. This study’s findings suggest that increased awareness and consumption of low-lactose milk could be a viable strategy for maintaining bone health in individuals with self-reported LI in Japan. This approach is supported by the observation that individuals with self-reported LI often exhibit low calcium intake and reduced BMD. Promoting the benefits of low-lactose milk, which is less likely to trigger LI symptoms, may contribute positively to this demographic’s nutritional health.

The strength of this study is the use of large amounts of medical checkup data from residents in Japan. This enabled the analysis to be performed with participants using real-world data. However, this study has some limitations. First, the IHPP targeted a rural population with a higher mean age, which included fewer young adults than the average of the Japanese population, resulting in a potential selection bias for age. Second, only the BMD data of the radius were available for IHPP. The BMD of the femoral neck and lumbar spine is considered the gold standard for the diagnosis of osteoporosis. However, this study was conducted as part of a medical checkup for general residents, and owing to the limitations of cost and space, the BMD of the femoral neck or lumbar spine could not be measured [25]. Third, this study focused on self-reported LI as it relates directly to consumption behavior. It is important to note that definitive diagnostic tests for lactose intolerance, such as hydrogen breath tests, were not conducted; thus, self-reported LI did not completely align with medically defined LI. Finally, causality was not clarified due to the nature of cross-sectional analyses. Further studies are warranted to demonstrate the causal relationships among self-reported LI, calcium intake, and BMD. Also, this study did not examine the effect of low-lactose milk. Further investigations are required to assess the efficacy of low-lactose milk consumption for individuals with self-reported LI.

## Conclusion

Self-reported LI is associated with low milk and calcium intake in healthy Japanese adults. Individuals with self-reported LI have a significantly lower BMD Z-score than those with non-self-reported LI. Moreover, low BMD T-scores are associated with self-reported LI after propensity score matching. Improved awareness and consumption of low-lactose milk provides a promising option to supplement calcium for self-reported LI. These findings focus on improving dietary management for bone health and highlight the potential benefits of dairy products, including low-lactose milk.

## Supplementary Information

Below is the link to the electronic supplementary material.

Supplementary Material 1

Supplementary Material 2

Supplementary Material 3

## Data Availability

The data, including analytical codes, cannot be publicly shared owing to ethical concerns. Data are available from the Hirosaki University COI Institutional Data Access/Ethics Committee (contact via e-mail: coi@hirosaki-u.ac.jp) to researchers who meet the criteria for data access. The researchers must be approved by the research ethics review board of the organization in which they are affiliated.
