# Supplementary material for: Self-reported lactose intolerance is inversely associated with calcium intake and bone mineral density: a cross-sectional data analysis from the Iwaki Health Promotion Project
Source: Eur J Nutr. 2025 Dec 6;65(1):4. doi: 10.1007/s00394-025-03856-x (PMC12681473; doi:10.1007/s00394-025-03856-x)
Supplement: Supplementary file 1 — Supplementary Material 1 [file 394_2025_3856_MOESM1_ESM.pdf]

Self-reported lactose intolerance is inversely associated with calcium intake and bone mineral density: A cross-sectional data analysis from the Iwaki Health Promotion Project

Daisuke Kawata[1,2*], Ayatake Nakano[1,2], Hiroshi M. Ueno[1,2], Yota Tatara[1,3], Eiji Sasaki[4], Yasuyuki Ishibashi[4], Yoshinori Tamada[1,5], Tatsuya Mikami[1,6], Koichi Murashita[1,7], Shigeyuki Nakaji[1], Ken Itoh[1,8]

European Journal of Nutrition

[1]Department of Precision Nutrition for Dairy Foods, Hirosaki University Graduate School of Medicine, Hirosaki, Japan
[2]Milk Science Research Institute, Megmilk Snow Brand Co., Ltd., Kawagoe, Japan
[3]Biomedical Research Center, Hirosaki University Graduate School of Medicine
[4]Department of Orthopaedic Surgery, Hirosaki University Graduate School of Medicine
[5]Department of Medical Data Intelligence, Research Center for Health-Medical Data Science, Hirosaki University Graduate School of Medicine
[6]Department of Preemptive Medicine, Innovation Center for Health Promotion, Hirosaki University Graduate School of Medicine
[7]Research Institute of Health Innovation, Hirosaki University, Hirosaki
[8]Department of Stress Response Science, Biomedical Research Center, Hirosaki University Graduate School of Medicine

[*]Correspondence: E-mail: daisuke-kawata@meg-snow.com

Supplementary table 1. Subgroup analysis of the association between self-reported LI and bone mineral density Z-score

Self-reported LI	Total					Male					Female				
Age group (years)	n	β	95% CI	*P*		n	β	95% CI	*P*		n	β	95% CI	*P*	
Under 45	297	-0.1737	(-0.438, 0.090)	0.196		119	0.4880	(-0.335, 0.554)	0.626		178	-0.3426	(-0.670, -0.015)	0.040	*
45 - 60	297	-0.2688	(-0.521, -0.017)	0.037	*	126	-0.4707	(-0.865, 0.077)	0.020	*	171	-0.0997	(-0.435, 0.236)	0.558	
Over 60	249	0.0105	(-0.283, 0.304)	0.944		128	-0.0477	(-0.453, 0.358)	0.816		121	0.0857	(-0.360, 0.531)	0.704	

Self-reported LI, self-reported lactose intolerance; CI, confidence interval; β, partial regression coefficient.
* $P<0.05$.

For each group, multiple regression analysis were conducted using adjustment factors of model 3 to examine the relationship between self-reported LI and bone mineral density Z-score.
The results of self-reported LI in each group are displayed in this table.
