# Supplementary material for: Self-reported lactose intolerance is inversely associated with calcium intake and bone mineral density: a cross-sectional data analysis from the Iwaki Health Promotion Project
Source: Eur J Nutr. 2025 Dec 6;65(1):4. doi: 10.1007/s00394-025-03856-x (PMC12681473; doi:10.1007/s00394-025-03856-x)
Supplement: Supplementary file 2 — Supplementary Material 2 [file 394_2025_3856_MOESM2_ESM.pdf]

Self-reported lactose intolerance is inversely associated with calcium intake and bone mineral density: A cross-sectional data analysis from the Iwaki Health Promotion Project

Daisuke Kawata[1,2*], Ayatake Nakano[1,2], Hiroshi M. Ueno[1,2], Yota Tatara[1,3], Eiji Sasaki[4], Yasuyuki Ishibashi[4], Yoshinori Tamada[1,5], Tatsuya Mikami[1,6], Koichi Murashita[1,7], Shigeyuki Nakaji[1], Ken Itoh[1,8]

European Journal of Nutrition

[1]Department of Precision Nutrition for Dairy Foods, Hirosaki University Graduate School of Medicine, Hirosaki, Japan
[2]Milk Science Research Institute, Megmilk Snow Brand Co., Ltd., Kawagoe, Japan
[3]Biomedical Research Center, Hirosaki University Graduate School of Medicine
[4]Department of Orthopaedic Surgery, Hirosaki University Graduate School of Medicine
[5]Department of Medical Data Intelligence, Research Center for Health-Medical Data Science, Hirosaki University Graduate School of Medicine
[6]Department of Preemptive Medicine, Innovation Center for Health Promotion, Hirosaki University Graduate School of Medicine
[7]Research Institute of Health Innovation, Hirosaki University, Hirosaki
[8]Department of Stress Response Science, Biomedical Research Center, Hirosaki University Graduate School of Medicine

[*]Correspondence: E-mail: daisuke-kawata@meg-snow.com

Supplementary table 2. Association of self-reported lactose intolerance with bone mineral density after propensity score matching adjusted with the use of supplements.

Objective variable	Z-score				T-score				Bone mineral density (g/cm^2)			
Variables	β	95% CI	*P*		β	95% CI	*P*		β	95% CI	*P*	
Self-reported LI (“Yes”=1, “No”=0)	-0.2557	(-0.442, -0.070)	0.007	**	-0.3112	(-0.548, -0.075)	0.010	*	-0.0149	(-0.028, -0.002)	0.023	*
Age (year)	-0.0031	(-0.010, 0.004)	0.395									
Age^2 /100 (year^2/100)					-0.0639	(-0.073, -0.055)	<0.001	***	-0.0035	(-0.004, -0.003)	<0.001	***
Sex (“Male”=0, “Female”=1)	0.5101	(0.292, 0.728)	<0.001	***	-0.1574	(-0.433, 0.122)	0.271		-0.1284	(-0.143, -0.113)	<0.001	***
BMI (kg/m^2)	0.0386	(0.011, 0.067)	0.007	**	0.0264	(-0.009, 0.062)	0.147		0.0018	(0.000, 0.004)	0.060	
Smoking status (current) (“Yes”=1, “No”=0)	-0.0495	(-0.322, 0.223)	0.721		0.0612	(-0.286, 0.408)	0.729		0.0014	(-0.017, 0.020)	0.883	
Smoking status (former) (“Yes”=1, “No”=0)	0.0457	(-0.181, 0.273)	0.693		0.2005	(-0.087, 0.488)	0.171		0.0109	(-0.005, 0.026)	0.170	
Alcohol consumption (g/1000kcal/day)	0.0114	(0.003, 0.020)	0.011	*	0.0139	(0.003, 0.025)	0.014	*	0.0007	(0.000, 0.001)	0.020	*
Vitamin D (μg/1000kcal/day)	-0.0125	(-0.061, 0.036)	0.616		-0.0245	(-0.087, 0.038)	0.441		-0.0011	(-0.004, 0.002)	0.519	
Walking speed (m/sec)	0.3701	(-0.128, 0.868)	0.145		0.1346	(-0.500, 0.769)	0.677		0.0157	(-0.019, 0.050)	0.368	
Use of calcium supplements (“Yes”=1, “No”=0)	0.1279	(-0.709, 0.964)	0.764		0.1832	(-0.881, 1.247)	0.735		0.0097	(-0.048, 0.067)	0.739	
Use of vitamin D supplements (“Yes”=1, “No”=0)	0.1040	(-0.494, 0.702)	0.732		0.1323	(-0.628, 0.892)	0.732		0.0071	(-0.034, 0.048)	0.735	
Const	-0.9339	(-2.037, 0.169)	0.097		0.9125	(-0.451, 2.276)	0.189		0.7945	(0.721, 0.868)	<0.001	***

Self-reported LI, self-reported lactose intolerance; CI, confidence interval; β, partial regression coefficient.
* *P*<0.05, ** *P*<0.01, *** *P*<0.001.
