# Supplementary material for: Self-reported lactose intolerance is inversely associated with calcium intake and bone mineral density: a cross-sectional data analysis from the Iwaki Health Promotion Project
Source: Eur J Nutr. 2025 Dec 6;65(1):4. doi: 10.1007/s00394-025-03856-x (PMC12681473; doi:10.1007/s00394-025-03856-x)
Supplement: Supplementary file 3 — Supplementary Material 3 [file 394_2025_3856_MOESM3_ESM.pdf]

Self-reported lactose intolerance is inversely associated with calcium intake and bone mineral density: A cross-sectional data analysis from the Iwaki Health Promotion Project

Daisuke Kawata[1,2*], Ayatake Nakano[1,2], Hiroshi M. Ueno[1,2], Yota Tatara[1,3], Eiji Sasaki[4], Yasuyuki Ishibashi[4], Yoshinori Tamada[1,5], Tatsuya Mikami[1,6], Koichi Murashita[1,7], Shigeyuki Nakaji[1], Ken Itoh[1,8]

European Journal of Nutrition

[1]Department of Precision Nutrition for Dairy Foods, Hirosaki University Graduate School of Medicine, Hirosaki, Japan
[2]Milk Science Research Institute, Megmilk Snow Brand Co., Ltd., Kawagoe, Japan
[3]Biomedical Research Center, Hirosaki University Graduate School of Medicine
[4]Department of Orthopaedic Surgery, Hirosaki University Graduate School of Medicine
[5]Department of Medical Data Intelligence, Research Center for Health-Medical Data Science, Hirosaki University Graduate School of Medicine
[6]Department of Preemptive Medicine, Innovation Center for Health Promotion, Hirosaki University Graduate School of Medicine
[7]Research Institute of Health Innovation, Hirosaki University, Hirosaki
[8]Department of Stress Response Science, Biomedical Research Center, Hirosaki University Graduate School of Medicine

[*]Correspondence: E-mail: daisuke-kawata@meg-snow.com

Supplementary Figure 1

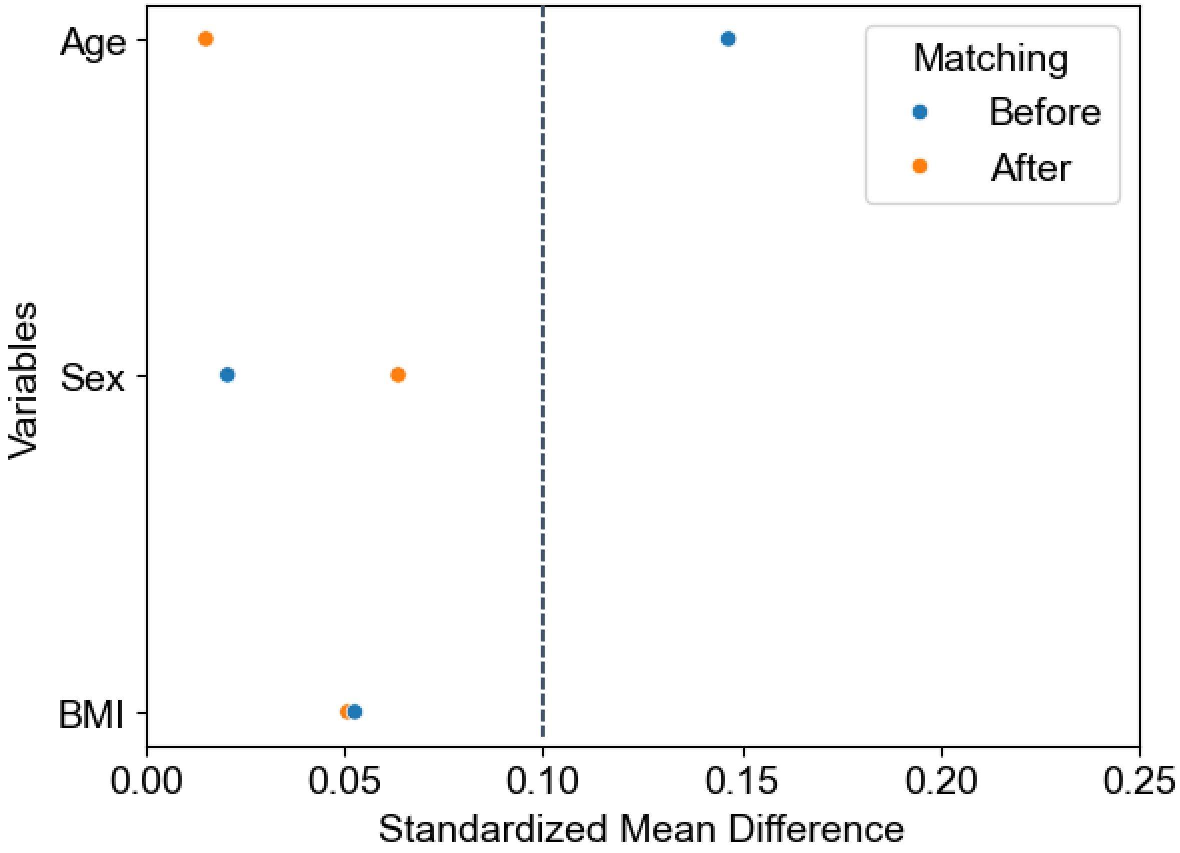

Standardized mean differences for each variable (Age, Sex, BMI) before and after propensity score matching were plotted (Before: blue, After: orange). Propensity score matching resulted in standardized mean differences less than 0.1 for all variables in the participants with and without self-reported lactose intolerance. BMI, body mass index.
